# The Wandsworth Guardians and Administrative Reform

**Published:** 1913-05-03

**Authors:** 


### THE WANDSWORTH GUARDIANS AND ADMINISTRATIVE REFORM.

### A Suggested Scheme of Centralised Control.

BY A LATE INFIRMARY MEDICAL OFFICER.

The resignation of the genial and trustworthy Medical Superintendent of St. John's Infirmary of the Wandsworth Union, affords an excellent opportunity to the Wandsworth Guardians of inaugurating a number of administrative medical reforms which on the whole would tend to a greater efficiency and administrative control of the institutional medical staff.

There are four large institutions in the Wandsworth Union the inmates of which require medical attention:

(1) St. John's Infirmary, where at present most of the chronic cases are treated, the only acute cases received being infants and lunatics. (2) The Tooting Annexe, where the epileptic, the consumptive, and the destitute aged of the Union are housed. (3) St. James' Infirmary, where most of the cases requiring surgical interference or medical treatment are forwarded, but in which there are at times a fair number of chronic cases, such as ulcers of the leg, etc. (4) The Workhouse and Branch Workhouse, where the maternity cases are conveyed.

At present there are two medical superintendents, each responsible to separate committees for the St. John's and St. James' institutions respectively; the medical officer of the workhouse, also responsible directly for his institutions; the resident medical officer of the Tooting Annexe is perhaps responsible to a part-time visiting officer, who is paid £200 a year for daily visits of two hours.

This arrangement of separate administrative heads is a cumbersome one, and the following scheme may be submitted for the consideration of the Wandsworth Guardians, the cost of the present arrangement being submitted for contrast.

Such a scheme would entail the adoption of certain other measures.

### The Suggested New Scheme.

(1) As noticed there is to be only one administrative head, appointed as medical superintendent of all the institutions. His salary must be definitely fixed and no extra fees allowed, the extra fees for lunacy or maternity cases or for notification being retained by the Guardians. (2) St. James' Infirmary to be made the *acute* institution, and in addition to its present work the lunacy cases, maternity cases, and the children to be transferred there. No maternity cases to be attended to in the workhouse. (3) St. John's Infirmary to be made the institution for the chronic cases, and only such cases as the medical superintendent directs to be transferred there. (4) No extra fees to be allowed to any of the medical officers. (5) The salaries of the senior residents to be fixed; the salaries of the junior resident to rise £10 to £15 annually. (6) Outside part-time appointments to be terminated, and if necessary the medical men involved compensated. No private practice or consulting practice to be allowed.

The advantages of such a scheme are: Centralisation under one responsible head, better organisation of the medical staff with better pay and prospects, and the treatment of all the acute cases in one institution, the avoidance of inter-institutional friction during transfers and disputes, and the making of St. James' Infirmary a school for general nursing training and maternity work.

The disadvantages of the present arrangement are the divided administration, the lack of co-operation, the probable collapse of St. John's Infirmary as a training school for nurses, and the compulsion of having to transfer maternity cases from a fairly well-equipped institution to the midwives in the workhouse. This is done because of some arrangement of midwifery fees, so much per case being due to the present non-resident medical officer of the workhouse, the fee apparently payable whether he is present at the birth or not.

It is perhaps too much to ask that these changes be inaugurated immediately. The schemes would cost about the same, the refunding of extra fees compensating any increased outlay. It is to be hoped that the Guardians will consider a revision of the present scheme and adopt one making for greater efficiency, greater control, and greater co-operation.

Proposed Scheme, with Probable Cost, etc.

| Office. | St. James' Infirmary. | St. John's Infirmary. | Tooting Annexe. | Workhouse. |
|---|---|---|---|---|
| | £ | £ | £ | £ |
| Medical Superintendent ... ... | — | 900 | — | — |
| Senior R. M. O. ... | 300 | 350 | — | — |
| Junior R. M. O., I. | 140 | 120 | 150 | 200 |
| " " II. | 120 | — | — | — |
| " " III. | 80 | — | — | — |
| | 640 | 470 | 150 | 200 |

= £900 + 1,460 = £2,360.

Present Arrangement, with Cost.

| Office. | St. James' Infirmary. | St. John's Infirmary. | Tooting Annexe. | Workhouse. |
|---|---|---|---|---|
| | £ | £ | £ | £ |
| Medical Superintendent ... ... | 450* | 500* | — | — |
| Senior R. M. O. ... | 180 | 175 | 140 | — |
| Junior R. M. O. ... | 120 | 120 | — | — |
| Outside Part-time Appointment ... | — | — | 200 | 350* |
| Assistant to last-named ... ... | — | — | — | 100 |
| | 750 | 795 | 340 | 450 |

= £2,335 + fees.
* + fees.

